# Ozone and Ozonated Oils in Skin Diseases: A Review

**DOI:** 10.1155/2010/610418

**Published:** 2010-07-04

**Authors:** V. Travagli, I. Zanardi, G. Valacchi, V. Bocci

**Affiliations:** ^1^Dipartimento Farmaco Chimico Tecnologico, Università degli Studi di Siena, Viale Aldo Moro 2, 53100 Siena, Italy; ^2^Dipartimento di Scienze Biomediche, Università degli Studi di Siena Viale Aldo Moro 2, 53100 Siena, Italy; ^3^Department of Food and Nutrition, Kyung Hee University, Seoul 130-761, Republic of Korea; ^4^Dipartimento di Fisiologia, Università degli Studi di Siena, Viale Aldo Moro 2, 53100 Siena, Italy

## Abstract

Although orthodox medicine has provided a variety of topical anti-infective agents, some of them have become scarcely effective owing to antibiotic- and chemotherapeutic-resistant pathogens. For more than a century, ozone has been known to be an excellent disinfectant that nevertheless had to be used with caution for its oxidizing properties. Only during the last decade it has been learned how to tame its great reactivity by precisely dosing its concentration and permanently incorporating the gas into triglycerides where gaseous ozone chemically reacts with unsaturated substrates leading to therapeutically active ozonated derivatives. Today the stability and efficacy of the ozonated oils have been already demonstrated, but owing to a plethora of commercial products, the present paper aims to analyze these derivatives suggesting the strategy to obtain products with the best characteristics.

## 1. Introduction

The increase of ageing, obesity, and diabetes in conjunction with inappropriate healthcare programs have emphasized the problem of having to treat almost 1.5 billion people affected by skin and mucosal infections due to bacteria, viruses, protozoa, and dysmetabolism. Pathologies range from the diabetic foot (ulcer with necrosis), bed sores, ulcers after a trauma or burns, chronic viral infections due to either herpes virus I and II, or human papylloma viruses, vaginal infections now frequent also in young girls due to *Candida*, *Trichomonas*, and *Chlamidia*, rectal mucosa infections such as anal ragadis, abscesses with fistula to end with mouth aphthous ulcers. These infections are rarely deadly but are considerably distressing because many patients often suffer of diabetes or vascular diseases with tissue hypoxia, other patients are immunosuppressed drugaddicts, or with concomitant HIV infection. Official medicine provides a variety of drugs that are expensive and often poorly efficacious because infections in hypoxic tissue contain methicillin-resistant *Staphylococcus aureus* and *Pseudomonas aeruginosa*. Patients are suffering not only because they become uncompliant to frequent medications but they are discouraged by observing a lack of healing [1]. Wound healing is a multiphase process involving blood clotting, inflammation, tissue proliferation, and remodelling [2], but both innate and adoptive immune systems are too often hindered by the chronic infection naturally difficult to overcome. This is also the reason explaining the failure of growth factors in heavily contaminated ulcers [3, 4].

The judicious use of ozone (O_3_) appears providential because first of all eliminates the pathogens and then, by releasing oxygen (O_2_), activates the proliferation of fibroblasts, hence the building of intercellular matrix with consequent proliferation of keratinoblasts and successive healing.

In Section 2, we propose to briefly review the physical chemistry of oil ozonation and all the basic analyses necessary for demonstrating the quality of the obtained products. In Section 3, it appears useful to inform readers that both skin and mucosae are sensitive to excessive amounts of gaseous O_3_ as there are clear demonstrations of a variety of alterations linked to a prolonged exposure. In Section 4, we will then clarify the various procedures devised to enhance the disinfectant and healing-promoting properties of O_3_. Finally, after an extensive analysis of a cornucopia of proposals, we will try to suggest guidelines for the future medical application of topical ozone and its derivatives (Section 5).

## 2. Physical Chemistry of Oil Ozonation with a Description of the Analytical Methods for Characterizing the Process

**Figure 1:**
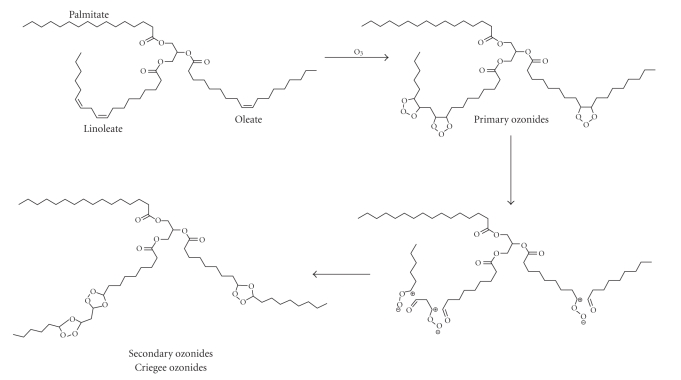
Representative chemical structures of ozonated derivatives which are formed by chemical reaction of ozone with unsaturated triglycerides. The primary ozonides are transient, unstable species which rearrange in the normal, secondary ozonides also known as Criegee ozonides.

Unsaturated lipid substrates react with insufflated gaseous O_2_/O_3_ mixture leading to therapeutically active ozonated derivatives (Figure 1).

Briefly, the postulated mechanism known as Criegee reaction provides that ozone combines with an unsaturated bond to form an initial, unstable primary ozonide which readily decomposes to form a zwitterions and a carbonyl fragment. In anhydrous environment these substrates combine to give the typical cyclic trioxolane derivative. 

However, the word “ozonated” is itself without scientific meaning if it is not associated with “how much” peroxides are present in the oil. In fact, from a therapeutic point of view, the ozonide compositions have the capacity to deliver active O_2_ and/or other useful species deep within the lesion without causing primary skin irritation. The few studies concerned with the therapeutic effects of ozonated oils on acute cutaneous wound healing in animal models do not investigate the dose/behaviour response, expressed as the amount of peroxides existing in the ozonated derivative used [5]. Recently, a quantitative evaluation of the therapeutic effect of topically applied ozonated sesame oil on acute cutaneous wound healing in mice as animal model has been developed [6]. The results indicate that both low (<1000) and high doses (>3000), as expressed in terms of peroxide value (see the corresponding section in this paper), delay cutaneous wound healing. Such an evidence is reinforced by a number of results between groups where the “middle” concentration (about 1500) has the most beneficial effect in accelerating the wound closure ratio.

From an industrial applicative viewpoint, the overall quality of ozonated derivatives depends upon several parameters, such as: (i) the type and the quality of ozone generators; (ii) the ozonation conditions, in terms of reactors and time, material type and amount, presence of water and/or catalyzers; (iii) the efficacy of the ozonizer, in terms of O_3_ concentration output, gas flow, gas carrier. As for the latter, the use of medical grade O_2_ instead of air is an important point to be considered; in fact, air feedstock (containing about 78% of nitrogen) used for the ozonation of unsaturated substrates could lead to the production of potentially toxic nitrated by-products [7], and to a significant decrease of the ozonation efficiency [8]. Another important feature is that ozonated oil has to be unequivocally characterized in terms of the species contents as well as the reaction kinetics. For these purposes, the knowledge of the physicochemical properties of ozonated vegetable oils during production has a great importance for their characterization and identification. For determining the quality of ozonated products, spectroscopic techniques, as Fourier-Transformed Infrared (FT-IR) and ^1^H and ^13^C-NMR [9], together with analytical methods as peroxide, acidity, and iodine values as well as viscometric determination are usually carried out [10].

### 2.1. FT-IR Spectroscopy

FT-IR spectroscopy is used to highlight differences in the functional groups during the oil ozonation, in particular the decrease of the bands corresponding to both C  =  C and =C–H stretching (e.g., sesame oil at 1654 cm^−1^ and 3009 cm^−1^, respe), and the increase of the band corresponding to ozonide CO stretching (e.g., sesame oil at 1105 cm^−1^). 

Ozonated samples can be analyzed using two different methods.

1. An adequate aliquot (usually about 2 *μ*L) of sample is deposited between two disks of KBr, avoiding air bubble formation, then the percentage transmittance or other suitable parameters are measured in the range 4000–800cm^−1^. Spectra are obtained setting the appropriate scan summations and minimal resolution (generally, 16 at 4 cm^−1^, resp.).
2. An adequate aliquot (usually about 2 *μ*L) of sample is dissolved in a suitable solvent (preferably chloroform) and then the solution is settled in the sample holder avoiding air bubble formation, then the transmittance (expressed as a percentage) or other suitable parameters are measured in the range 4000–800 cm^−1^. Spectra are obtained setting the appropriate scan summations and minimal resolution (generally, 16 at 4 cm^−1^, resp.).

### 2.2. NMR Spectroscopy

^1^H and ^13^C NMR spectroscopies are performed to obtain more information about the variation of the functional groups involved in the reaction of ozonation. Both the disappearance of the signals relative to protons and carbons on the double bond (e.g., in sesame oil 5.29 ppm, and various signals in the range 127.8–130.0 ppm, resp.) and the parallel appearance of a signal on the proton and carbon of 1,2,4-trioxolane (e.g., in sesame oil in the 5.11–5.08 ppm range, and 103.4–104.3 ppm range, resp.) are evidenced. Quantitative analysis can be performed by spectra normalized with respect to the integral areas of the OCH_2_ protons (glycerol) that remain constant during the whole process.

Spectra will be obtained using suitable instruments by solubilizing the ozonated sample in a proper solvent (preferably CDCl_3_). Particularly, an adequate aliquot (usually about 100 *μ*L) of sample is solubilised with 750 *μ*L of CDCl_3_ in a 5 mm NMR tube, then the analysis will be performed. To obtain quantitative data, it is sufficient to perform a ^1^H-NMR, while ^13^C-NMR essentially provides qualitative informations [9].

### 2.3. Iodine Value

The iodine value (IV) represents the quantity of iodine (in grams) that will react with the double bonds in 100 grams of sample. IV is determined according to the Pharmacopoeia monographs. The IV is calculated by means of the following equation:

(1) $$\text{I}{\text{V}}_{}=\frac{1.269\cdot \left(n_{1}-n_{2}\right)}{m},$$

where *n*_1_ is the volume in mL of thiosulphate solution (0.1 M) used for carry out a blank test, *n*_2_ is the volume in mL of thiosulphate solution (0.1 M) used for the titration and *m *the quantity, in grams, of substance. It is, therefore, a measure of the total number of double bonds present in the sample and for such a reason it is a chemical analysis useful for evaluating the decrease of double bonds during the oil ozonation process, giving information about the 1,2,4-trioxolane formation.

### 2.4. Acid Value

The acid value (AV) is an index that expresses, in mg, the quantity of potassium hydroxide required to neutralise the free acids presents in 1 g of the substance. The AV is calculated by means of the following equation:

(2) $$\text{AV}=\frac{5.610\cdot n}{m},$$

where *n* is the volume in mL of titrant and *m* the quantity, in grams, of substance.

It is representative of the acidity level of the product and it represents an index of the degradation by-products that could be formed during the ozonation process.

### 2.5. Peroxide Value

Peroxide value, (PV), is usually used as an indicator of the advancement and/or the control of the ozonation process because of its simplicity, rapidity, and low cost. Moreover, the PV may be adequate for the stability evaluation of vegetable oil ozonides and it appears to be very important for commercial distribution as well as for the determination of the better storage modalities. However, it had been necessary to standardize the methodology for a validated PV. 

In the present paper, a detailed analysis of PV assessments of ozonated lipid derivatives based on both literature data and our laboratory experiments will be presented together with their possible correlations with other techniques. Such a report allows an in-depth acquaintance of the ozonation process of vegetable oils as well as of the related products obtained, allowing to define the quality parameters useful for industrial purposes. Specifically, the peroxide value (PV) represents the quantity of peroxide expressing in milliequivalents of active O_2_ contained in 1000 g of the sample.

For the PV evaluation, three different methods were adopted.

- First official monograph described in Pharmacopoeia (e.g., European Pharmacopoeia, British Pharmacopoeia, United States Pharmacopoeia), which provides the solubilization of sample in 30 mL of chloroform/glacial acetic acid (2 : 3), the addition of saturated potassium iodide solution (0.5 mL) and the titration after 1 minute with a solution of sodium thiosulphate.
- Second method described by Martinez Tellez et al. [11], which always provides the solubilization of sample in 30 mL of chloroform/glacial acetic acid (2 : 3) and the addition of saturated potassium iodide solution (0.5 mL), but the titration is done after 24  hours.
- Third method recently proposed [10]. Briefly, 2 g of SO were weighed in a 250 mL conical flask and 30 mL of chloroform/glacial acetic acid (2 : 3) were added. Then, 3.0 mL of saturated potassium iodide solution were added. The flask was stirred at reflux temperature (60°C) for various times (5–180 minutes). After this time, the solution was cooled and 25 mL of water were added. Solutions of sodium thiosulphate at the appropriate concentration (0.0001–0.1 M) were used for the titration.

In all determinations the PV was calculated by means of the following equation:

(3) $$\text{PV}=\frac{1000\cdot \left(V_{1}-V_{0}\right)\cdot c}{m},$$

where *V*_1_ is the volume in mL of thiosulphate solution used for the titration, *V*_0_ is the volume in mL of thiosulphate solution used for carry out a blank, *c* the thiosulphate concentration and *m* the sample quantity (grams).

The ozonation efficiency (expressed as a percentage) represents ratio of the amount of peroxidation due to ozonation process, as estimated by PV value, to the O_3_ total amount applied to the system. It was calculated by means of the following equation:

(4) $$\text{OE}=\frac{\left(\text{P}{\text{V}}_{\text{s}}-\text{P}{\text{V}}_{0}\right)}{1000}\times \frac{24}{\text{OAD}}\times 100,$$

where PV_s_ is the ozonated sample PV, PV_0_ is the PV of untreated sample, and OAD stands for the O_3_ applied dose (mg/g).

### 2.6. Viscosity Measurement

Viscosity evaluation is a useful technique because it is fast and it could be online, giving an estimation of the double bonds present in the sample. In fact, the greater the ozonation time the higher the product viscosity because of the disappearance of the double bonds. Moreover, its typical trend can be a useful tool in providing a rapid quality control assessment during the entire ozonation process, as well as to decide on the process time for obtaining the desired ozonation level of the sample [9].

## 3. Cutaneous Responses to Environmental Ozone Exposure

The skin, along with the respiratory tract, is directly exposed to environmental pollutants including O_3_, an important constituent of photochemical smog. Although numerous studies have documented effects of O_3_ on the respiratory tract in animals and humans [12–15], only recently some studies characterizing its effect on cutaneous tissue have been published [16–20]. The skin consists of two main layers, the inner dermis, mainly composed of fibroblasts and connective tissue matrix, and the outer epidermis, which contains keratinocytes that, by progressively differentiating to form enucleate corneocytes, become imbedded in a lipid matrix and together comprise the outermost part of the epidermis, the stratum corneum (SC) [21, 22].

Previous studies have shown that exposure to O_3_ results in the depletion of both water soluble and lipophilic antioxidants such as uric acid, ascorbic acid, and tocopherol, and this was accompanied by increase in parameters of both lipid peroxidation and protein modification, primarily in the outermost skin layers [16, 17, 23].

In further studies, we were also able to show that the exposure of hairless mice to O_3_ will not only deplete the antioxidant levels and increase oxidative markers but these molecules are able to induce active cell responses.

These effects can be briefly summarized as follows.

**(1) Induction of Redox Sensitive Transcription Factors**

Ozone, like many others environmental challenges, is able to activate transcriptional factors redox sensitive such as Nuclear Factor k B (NFkB). This transcriptional factor acts as an activator for a multitude of proinflammatory genes (IL-8, TNF*α*, TGF*β*) and adhesion molecules (ICAM and VCAM). It has been assessed that O_3_ is able to activate NFkB using both *in vitro* and *in vivo* systems. Thiele et al. [16], using an immortalized human keratinocytes (HaCaT cells), were able to show that O_3_ induced the activation of NFkB by electrophoretic mobility shift assay (EMSA). Ozone induced a dose dependent activation of the transcription factor. This effect was likely to be mediated by ROS, particularly H_2_O_2_, because it was inhibited by the incubation of the cells with lipid soluble antioxidants (tocopherol).

**(2) Induction of Heat Shock Protein (HSP) and Inflammatory Markers**

As a consequence of the induction of transcription factors, O_3_ exposure (6 days to 0.8 *μ*g/mL for 6 hours/day) induced the expression of proinflammatory markers in skin homogenates such as cyclooxygenase-2 (COX-2). This induction was accompanied by an increase level of heat shock protein (HSP) 32, also known as heme oxygenase-1 (HO-1). In this paper, we were the first to demonstrate the upregulation of HSPs 27, 32 and 70 in homogenized murine skin upon O_3_ exposure. HSP27 showed the earliest (2 hours) and highest (20-fold) response to O_3_ compared with the delayed induction (12 hours) of HSP70 and HO-1. HSP27 is expressed predominantly in the suprabasal epidermis in human skin, whereas HSP70 predominates in the dermis compared with the epidermis. These differences in location between HSP27 and HSP70 might explain the different time course of induction of these stress proteins upon O_3_ exposure. It is therefore possible that the generated bioactive compounds may be responsible for the induction of HSPs as was also shown after UV irradiation.

**(3) Induction of Matrix Metalloproteinases (MMPs)**

Among the multiple systems altered in the skin by environmental pollutants, MMPs are among the major targets. Indeed, O_3_ exposure is able to affect their synthesis and/or activity with logical consequences on tissue remodeling and wound healing [23, 24]. Within the MMP family, MMP-2 and MMP-9 are the only members able to degrade type-IV collagen of the basal membranes [25]. MMP-2 is involved in pathological processes such as photoageing and precancerous/cancerous skin lesions after UV exposure; moreover, MMP-2 is capable of cleaving other substrates, in addition to type-IV collagen, including other MMPs and therefore can (indirectly) control extracellular matrix degradation and remodelling.

MMP-9, like MMP-2, plays a role in human skin ageing [26] tumor development [27], as well as in other cutaneous lesions such as psoriasis and dermatitis [28, 29]. In a recent study, we were able to demonstrate that O_3_, was able to affect MMP activity. Most likely the generation of bioactive molecules can be the cause of such activation. It has been also demonstrated that O_3_ is able to induce NO production via the activation of iNOS in cutaneous tissues [18]. When produced in excess, NO, may combine with superoxide to form peroxinitrite (derived from other sources) that can activated MMPs especially MMP-9. Thus, the increase of oxidative stress after O_3_ exposure, plus the interaction between O_2_ and nitrogen active molecules might be the main mechanism that leads to the enhanced MMPs activities in skin tissues. This can be also a result from an imbalance between MMPs and their endogenous inhibitors, the tissue inhibitors of metalloproteinases (TIMPs) [30].

In fact, the activities of MMPs are regulated by TIMPs, which can be produced by a multitude of cell types present in the cutaneous tissue. While MMP activity is altered by the O_3_, neither TIMP-1 nor TIMP-2 level expression is affected. The lack of changes in TIMP-1 and 2 levels, combined with the increased activity of MMPs suggest that O_3_ can cause a net increase in matrix degradation. On the other hand, in a comparative study where normal skin has been exposed for two hours to environmentally realistic levels of ozone, only a moderate state of oxidative stress at level of the stratum corneum has been induced, without producing a visible clinical response [31].

## 4. Skin Age-Related Responses to Ozone Exposure: Wound Healing

Wound healing is a critical process in the skin and it has known to be affected by oxidative stress and also to decline with increasing age [32]. Although the exact sequence of wound healing is complex, cutaneous wound healing begins with wounding induced signaling factor-based transformation of stationary keratinocytes into cells capable of both replication and migration. Upon transformation, these cells express a host of molecules that promote the invasion of the injured epithelial matrix and reepithelialisation of the wound surface [33]. Delayed wound healing in the elderly has been well described [34]. 

As mentioned above, O_3_ exposure is also associated with activation of transcription factor NFkB, which is important to regulate inflammatory responses and eventually entire wound healing. O_3_ exposure increased levels of Transforming Growth Factor (TGF-*β*) that is a critical factor in tissue remodeling [35, 36]. We can summarize that while O_3_ as an oxidant, might stimulate wound healing, it would be detrimental in an “aging environment” due to the increased concentration-dependent oxidative stress. Therefore, these aspects have biological as well as practical implications and needed further investigations. 

In a recent study, we demonstrated the detrimental effects of O_3_ on cutaneous wound healing in the aged animals. In fact, when hairless young (8-week-old) and aged mice (18-months-old) with after full thickness excisional wounds were exposed to 0.5 *μ*g/mL O_3_ for 6 hours per day the rate of wound closure was significantly delayed in the old group. We also showed induction of protein and lipid oxidation assessed as changes in protein oxidation (carbonyls) and lipid peroxidation (4-hydroxynonenal, HNE adducts) in the old mice compared to the young mice during the later stage of cutaneous wound healing. O_3_ exposure has different effects depending on the age of the mice. In fact, it significantly delayed wound closure in old mice, while in young mice, it led to accelerated trend during the first few days of the exposure. This might be attributed to the antibacterial properties of O_3_, as it has been shown that application of “hydropressive” ozonation provides fast cleansing of wound surface from pyonecrotic masses, promotes elimination of infection and thus substantially reduces the period of treatment of the patients [37]. Recently, clinical treatments using hyperbaric oxygen therapy demonstrated that increased O_2_ tension at the wound site increases the formation of granulation tissue, enhances accelerated wound closure and ameliorates impaired dermal wound healing [38]; therefore, accelerated trend of wound closure shown in young population may be due to decreased bacterial infection and/or increased O_2_ tension by O_3_ exposure in wound area. 

One of the possible driving processes of the effect of O_3_ on wound healing can be also in this case the modulation of the transcription factor NFkB. Interestingly, the dose–effect relationship between level of oxidative stress and NFkB exhibits a biphasic profile: while moderate levels of oxidative stress activate NFkB through an IkB kinase independent mechanism, extremely high levels of oxidative stress have been shown to inhibit NFkB activation by blocking IkB*α* phosphorylation [39]. One potential explanation for the differential effect in the older animals is that the level of oxidative stress generated by O_3_ exposure combined with aging causes levels of oxidative stress that inhibits IkB*α* phosphorylation, thereby resulting in a decline in NFkB activation. This finding is consistent with what mentioned previously that O_3_ exposure induced skin antioxidants depletion. 

This interpretation is also bolstered by data on TGF-*β* a crucial modulator of tissue remodeling and is linked to both NFkB status as well as to levels of oxidative stress during entire wound healing process [40]. The reduced TGF*β* levels in both air and O_3_ exposed old mice as well as the lower induction of TGF*β* by O_3_ exposure in the old animals suggests that the noted delays in wound closure might be related to defects in oxidative stress-dependent NFkB status as well as levels of oxidative stress and TGF*β* signaling in aged mice during later stage of wound healing.

## 5. Topical Application of Ozone in Medicine

To the best of our knowledge, the first application of gaseous O_3_ was performed during World War I for treating German soldiers affected by gaseous gangrene due to Clostridium anaerobic infections very sensitive to O_3_ [41, 42]. In 1936, Dr P. Aubourg, by using a metal cannula, was the first to propose the insufflations of gaseous O_2_/O_3_ in the rectum to treat chronic colitis, anal ragadis and fistulae. This approach is very empirical and unprecise and today it is mostly used by Cuban physicians. In 1937, a Swiss dentist, E. A. Fisch (1899–1966) had the idea to use it in his practice and, by a twist of fate, he treated Dr. E. Payr (1871–1946) a surgeon who had a painful gangrenous pulpit. Payr was so enthusiastic of the O_3_ effect to use it in his surgical practice with great advantage [43]. Later on, Werkmeister [44] mastered the use of gaseous O_3_ in several skin ulcers due to atherosclerosis, diabetes and radiotherapy by either enclosing a leg in a polythene-bag (the so-called bagging system) or using an ozone-resistant plastic cup applied in other areas. In the former application the gas was introduced to just inflate the bag containing some distilled water. The system was static but after 20–25 minutes the gas was aspirated and destroyed. The O_3_ concentrations varied between a high 80 *μ*g/mL in very purulent ulcers and progressively lower concentrations down to 10 *μ*g/mL as the ulcers improved because excessive O_3_ would be deleterious for healing. As the cup system had an inlet and an outlet, Werkmeister could realize a continuous gas flow with a modest depression that enhanced the vasodilation of the ulcer's area. With both systems he treated many extensive and otherwise incurable lesions within 50–200 days. It is noteworthy that gaseous O_3_ works well only in a water vapour-saturated bag because it must dissolve into superficial water or in the exudate to react proficiently. The normal skin does not undergo any damage during the treatment. Today these procedures are still in use but they are somewhat cumbersome and great care must be exercised to prevent air contamination.

How ozonated oils act remains an open question. Probably, when the stable triozonide comes into contact with the warm exudate of the wound, it slowly decomposes into different peroxides, which readily dissolves in water, probably generating hydrogen peroxide that can explain the prolonged disinfectant and stimulatory activity. If it is correct, this reasoning implies that we should have titrated preparations with high, medium, or low ozonide concentrations to be used during the inflammatory septic phase I, regenerating phase II or remodelling phase III, respectively [2]. These phases have been related to the rapidly changing cell types and to the release of cytokines and growth factors that modulate the complex healing process.

An alternative method for treating diabetic foot ulcers is the use of hyperbaric oxygen therapy (HOT) but in such a case one disadvantage is the use of only hyperbaric O_2_ and another is the need to close the patient in the chamber for two hours. Therapeutic results are far more modest than topical O_3_ application, particularly when it is contained in a close cabinet with thermostatically-controlled temperature. However this procedure requires considerable idle times and, if an aspirating pump is unavailable, it may contaminate the operating room. For these reason today for cleaning and disinfecting cutaneous and mucosal infections and lesions due to many causes (like, e.g., trauma, ischemia, burns), it appears preferable to use at once freshly ozonated water and then ozonated oil, particularly during the night or at rest conditions. 

The process of water ozonation needs of double distilled water and O_3_ concentrations ranging from 20 up to 100 *μ*g/mL of gas to have a final yield of 5 up to 25 *μ*g/mL, respectively. O_3_ is directly bubbled into the water and the gas in excess is passed through a dehydrating device and finally through a destructor. Depending upon the water volume and the gas flow, a period of ozonation between 5–20 minutes is sufficient to saturate the water with gaseous O_3_. In fact, if the water is ultrapure, O_3_ physically dissolves in the absence of chemical reactions and if kept in a glass bottle closed with a Teflon cap, the concentration halves only after 300 hours at 0°C. However, at 20°C the half-life is about 10 hours [45]. It must be noted that monodistilled water allows a much faster O_3_ decomposition and it is not practical. It is adviced to maintain the bottle at 4°C and to quickly close the bottle at any time, or better to have a valve system to prevent gas losses. It would be useful to device a procedure for maintaining the O_3_ concentration for longer times and we are investigating a possible procedure. On the other hand, ozonation of either olive or sunflower oils requires a much longer time and the procedure needs to be well-standardized in terms of gas-flow, O_3_ concentration, oil volume, and temperature. As recently reviewed, at least twenty different vegetable oils have been patented but so far it remains impossible to define their relative cost/benefit [46]. At this stage, after evaluating several physicochemical criteria, stability, efficacy, and cost, it seems that sesame oil has several advantages in comparison to other oils. 

How and when ozonated water and oils are used? Chronic wounds range from diabetic foot to putrid and deep ulcers due to limb atherosclerosis, or trauma and burns. Moreover, both immunosuppressive chemotherapy and/or malnutrition cause abscesses, anal fissures and fistulae, bed sores, furunculosis, and osteomyelitis which are difficult to treat and often fail after prolonged treatments. About 7 million patients in the United States are affected with a cost over US$ 25 billion annually. Various types of disinfectants, antibiotics, antifungal, antiprotozoal, and growth factors are scarcely effective because the deranged metabolism and local hypoxia are not modified. Several other approaches such as vacuum therapy [47, 48], maggot therapy [49] and devices for providing topical oxygen therapy in a clinical setting have been proposed and variably used. This last approach has a rationale in the sense that enhanced oxygenation is useful for activating the metabolism and cell proliferation of ischemic tissues [50–52]. However, it has also considerable limitations because it is a cumbersome therapy, with minimal disinfectant activity and modifications of the fundamental pathogenetic mechanisms.

Another topic of critical interest is the pathologies of the vaginal mucosa. Although rarely deadly (as the toxic shock syndrome due to a forgotten absorbent tampon), a majority of women physically and psychologically frequently suffer from a number of infections due to several pathogens such as Neisseria gonorrhoeae, Trichomonas vaginalis, Candida albicans, Chlamidia trachomatis, Herpes virus type-II (HV-II), human papilloma viruses (HPV), human immunodeficiency virus (HIV), often due to unprotected sexual intercourses, stress, change in sexual partners and also physiological hormonal changes during menopausa. About 20 million Americans are affected by the distressing HV-II and as many 40 million have the genital HPV with warts and the impending risk of cervix cancer. Moreover, the further implantation of opportunistic infections complicates the treatment. It is unfortunate that orthodox medications are expensive and not so useful because of drug-resistant pathogens and side effects limiting the compliance. So far official medicine has not yet entertained the topical use of O_3_ and derivatives in therapy because they are not profitable and no extensive clinical trials have been published in peer-reviewed journals: the therapy has remained in practitioners' hands and the results remain anecdotal. Moreover, the parenteral use of ozone, also known as ozone therapy, is very useful as adjuvant: it is reasonably ease to perform in terms of classical ozonated major and minor autohemotherapy [53]. The latter modality has been successfully used for eliminating recurrences of HV-I and II infections. However, topical therapy is essential and it is carried out by using vaginal irrigation of fresh ozonated water and application of vaginal ozonated oil pessaries for the night. During prolonged treatment the ozonated compounds allows the elimination of any pathogens. So far no resistance to O_3_ has been demonstrated. Creams containing ozonated oils can be used 3-4 times daily for external genital areas and also for several anorectal affections. 

As for the oral infections (aphthae, HV-I, opportunistic superinfections, or acne) the earliest as possible application of ozonated ointments, by minimizing pathogen diffusion and enhancing microcirculation, reduces the swelling, destroys the pathogen, and allows a rapid healing. 

Last but not least, clinical trials in tinea pedis as well as onychomycosis [54, 55] have been recently published and have shown the usefulness of ozonated sunflower oil.

## 6. Conclusions

At the present, especially in young people, venereal infections are increasingly frequent and therefore a suitable, effective medication with ozonated compounds will be a huge economical and social value. Also, elderly people are burdened with a variety of wounds and ulcers, some of which never heal, making life miserable. It is hoped that the present paper will inform official medicine for this advance and will incite to programme suitable clinical trials to show the full efficacy of ozone therapy by evidence-based medicine.
